# Enumeration Approach to Atom-to-Atom Mapping Accelerated by Ising Computing

**DOI:** 10.1021/acs.jcim.4c01871

**Published:** 2025-02-02

**Authors:** Mohammad Ali, Yuta Mizuno, Seiji Akiyama, Yuuya Nagata, Tamiki Komatsuzaki

**Affiliations:** † Graduate School of Chemical Sciences and Engineering, Hokkaido University, Kita 13, Nishi 8, Kita-ku, Sapporo 060-8628, Hokkaido, Japan; ‡ Statistics Discipline, Khulna University, Sher-E-Bangla Road, Khulna 9208, Bangladesh; § Research Institute for Electronic Science, Hokkaido University, Kita 20, Nishi 10, Kita-ku, Sapporo 001-0020, Hokkaido, Japan; ∥ Institute for Chemical Reaction Design and Discovery, Hokkaido University, Kita 21, Nishi 10, Kita-ku, Sapporo 001-0021, Hokkaido, Japan; ⊥ ERATO Maeda Artificial Intelligence for Chemical Reaction Design and Discovery Project, Hokkaido University, Kita 21, Nishi 10, Kita-ku, Sapporo 001-0021, Hokkaido, Japan; # SANKEN, Osaka University, 8-1 Mihogaoka, Osaka 567-0047, Ibaraki, Japan

## Abstract

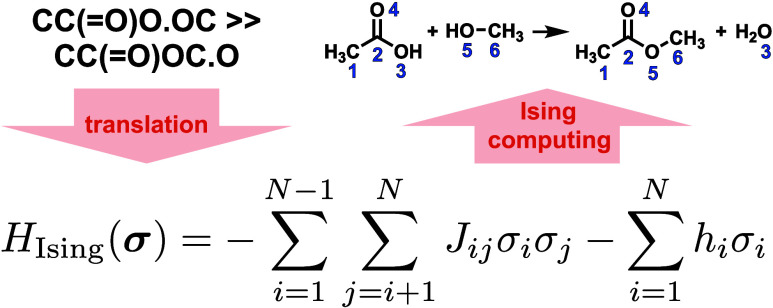

Chemical reactions are regarded as transformations of chemical structures, and the question of which atoms in the reactants correspond to which atoms in the products has attracted chemists for a long time. Atom-to-atom mapping (AAM) is a procedure that establishes such correspondence(s) between the atoms of reactants and products in a chemical reaction. Currently, automatic AAM tools play a pivotal role in various chemoinformatics tasks. However, achieving accurate automatic AAM for complex or unknown reactions within a reasonable computation time remains a significant challenge due to the combinatorial nature of the problem and the difficulty in applying appropriate reaction rules. In this study, we propose a rule-free AAM algorithm, which enumerates all atom-to-atom correspondences that minimize the number of bond cleavages and formations during the reaction. To reduce the computational burden associated with the combinatorial optimization (i.e., minimizing bond changes), we introduce Ising computing, a computing paradigm that has gained significant attention for its efficiency in solving hard combinatorial optimization problems. We found that our Ising computing framework outperforms conventional combinatorial optimization algorithms in terms of computation times, making it feasible to solve the AAM problem without reaction rules in an acceptable time. Furthermore, our AAM algorithm successfully found the correct AAM solution for all problems in a benchmark data set. In contrast, conventional AAM algorithms based on chemical heuristics failed for several problems. Specifically, these algorithms either failed to find the optimal solution in terms of bond changes, or they identified only one optimal solution, which was incorrect when multiple optimal solutions exist. These results emphasize the importance of enumerating all optimal correspondences that minimize bond changes, which is effectively achieved by our Ising-computing framework.

## 1 Introduction

Atom-to-atom mapping (AAM)^1^ is a procedure that establishes a one-to-one correspondence between the atoms in the reactants and products of a chemical reaction. AAM provides the information on the chemical structure transformation, including the identification of bonds broken and formed, as well as reaction centers. This information is crucial for various applications, such as retrosynthesis planning,^2−4^ reaction classification,^5^ chemical reaction searching,^6−9^ as well as clarifying mechanisms of enzymatic reactions or discovering metabolic pathways.^1,10,11^ Consequently, AAM plays a significant role in developing chemical reaction databases,^12,13^ which are used extensively in chemoinformatics research.

AAM has been accomplished through manual curation by expert chemists or automatic algorithms. However, manual AAM is a labor-intensive and time-consuming process that relies on expert chemical knowledge, so it becomes impractical as the complexity and scale of chemical data sets increase. Thus, automated approaches have become prominent.

Despite the availability of numerous AAM algorithms and tools,^1,10,11,14−20^ accurate automatic AAM for complex or unknown reactions remains challenging. In existing approaches, AAM is typically formulated as an NP-hard combinatorial optimization problem, such as finding maximum common subgraphs.^17^ Consequently, the computation time required to identify exactly optimal mapping(s) increases exponentially with the number of atoms involved in a chemical reaction. Due to the NP-hard nature, existing AAM frameworks often rely on heuristics, such as incorporating known chemical rules^14,16^ and machine learning techniques.^19,21−23^ However, the criteria for selecting the reaction rule sets are strongly dependent on the individual data set,^14,16^ and identifying the correct reaction rules for various reaction cases and data sets is still challenging. Furthermore, the accuracy of the rule-based automatic AAM for reaction types not included in the data set may be low due to the lack of reaction rules. Machine learning-based approaches sometimes show incorrect mapping based on the frequently appearing reaction patterns in the training data set.^23^ Therefore, accurate automatic AAM demands a universal algorithm that relies on minimal chemical knowledge, without the need for known reaction rules or training data, particularly for unknown reactions.

Moreover, chemical reactions may have multiple potential AAM solution candidates. In such ambiguous cases, the chemically correct mapping based on the actual reaction mechanism is typically determined through experiments, such as isotope labeling.^24,25^ Therefore, an exhaustive enumeration of potential candidate mappings is an effective approach to identifying all probable reaction patterns in unknown reactions. Based on these candidates, experimental chemists can clarify a mechanism by a designed experiment to distinguish these candidates. It is important to note that such multiple candidate mappings frequently include chemically “equivalent” transformations, which arise due to molecular symmetry in the reaction.^1,22,26^ Thus, to effectively identify all distinct reaction patterns represented by “nonequivalent” mappings, a symmetry reduction method is required.

We propose an AAM algorithm that enumerates all plausible mappings without reaction rules. We formulate the AAM problem as maximum common edge subgraph (MCES) problem,^27,28^ which corresponds to finding atom label mapping(s) that minimize the number of bond cleavages and formations. The MCES problem is then reduced to the maximum clique problem, i.e., finding the largest subgraph(s) where all nodes are fully connected to each other. After enumerating all maximum cliques, candidate mappings are constructed and then clustered into distinct reaction patterns through a symmetry reduction method. We elaborate on our proposed framework in Section 2.

To solve the maximum clique problem efficiently, our proposed framework employs an enumeration algorithm utilizing Ising computing.^29^ Ising computing is a computational paradigm that uses Ising models (or spin glass models), which are statistical mechanics models of magnetic alloys. Seminal studies on Ising computing include the development of the Hopfield network^30,31^ with its application to combinatorial optimization,^32^ the Boltzmann machine,^33^ and simulated annealing.^34^ The Hopfield network and the Boltzmann machine have been successfully applied to graph matching,^35^ approximating maximum cliques^36^ and chemoinformatics tasks such as substructural search,^37^ spatial molecular alignment,^38,39^ and similarity search.^40^ Simulated annealing (SA) forms another research stream in Ising computing for combinatorial optimization, including quantum annealing^41^ and its physical realization,^42^ as well as their applications to chemistry.^43,44^ Currently, various types of Ising computing have been proposed and implemented,^45^ gaining significant attention as efficient methods for solving hard combinatorial optimization problems. In our framework, the maximum clique problem is formulated as an Ising model problem and solved using SA. Additionally, we employ the enumeration algorithm^29^ that utilizes SA to sample maximum cliques. This enumeration algorithm repeats sampling to collect all maximum cliques, with an appropriate stopping criterion for sampling, derived based on probability theory. This sampling-based enumeration approach is the main point that differentiates our AAM framework from the other Ising computing approaches mentioned above.

We applied our proposed AAM algorithm to a benchmark data set of AAM.^16^ We found that the enumeration algorithm using Ising-computing outperforms a conventional exact algorithm for the maximum clique problem in terms of computation time. Additionally, our algorithm successfully found the correct mappings in all reactions of the benchmark data set. We also clarified in some reactions that the enumeration of multiple mappings is especially significant in covering correct mappings without any reaction rules. The benchmark results and discussion are detailed in Section 3.

## 2 Proposed Algorithm

**Figure 1:**
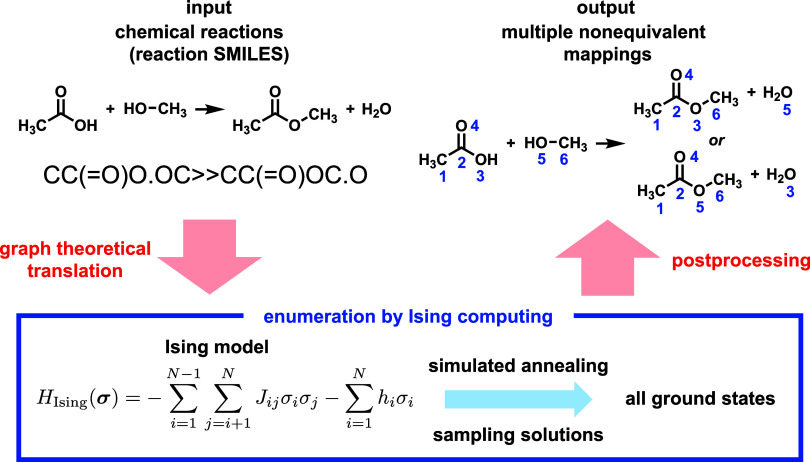
Overview of our proposed algorithm.

Figure 1 illustrates our algorithm for enumerating all plausible, nonequivalent mappings of a chemical reaction. Through graph theoretical reduction, this algorithm transforms the AAM problem into a maximum clique problem, formulated as a quadratic unconstrained binary optimization (QUBO) problem, which is equivalent to an Ising model problem. The maximum clique enumeration problem is subsequently solved by the enumeration algorithm using Ising computing.^29^ In this study, we specifically employ simulated annealing (SA)^34^ for this purpose. Each maximum clique found corresponds to a set of chemical bonds that remain unchanged during the reaction. Based on these unchanged chemical bonds, subsets of reactant atoms are mapped to subsets of product atoms, which we refer to as partial mapping. Then, the algorithm permutes the residual atoms not included in each partial mapping to generate all plausible complete mappings. Finally, these complete mappings are clustered by the molecular symmetry to identify all possible nonequivalent mappings, which can result in distinguishable reaction mechanisms. Additionally, several optional filters may be applied to reduce the number of plausible nonequivalent mappings. In the following subsections, we will detail this procedure.

### 2.1 Translating the AAM Problem into the Maximum Clique Problem

The AAM problem is typically modeled as the maximum common edge subgraph (MCES) problem between the molecular graphs of reactants (*R*) and products (*P*).^46,47^ The MCESs between *R* and *P* are the common substructures that maximize the number of edges (bonds) shared by the reactant and product graphs. In other words, the MCESs consist of chemical bonds that likely to be preserved during the reaction, maximizing the number of such unchanged bonds (or equivalently, minimizing the number of bonds broken or formed).

**Figure 2:**
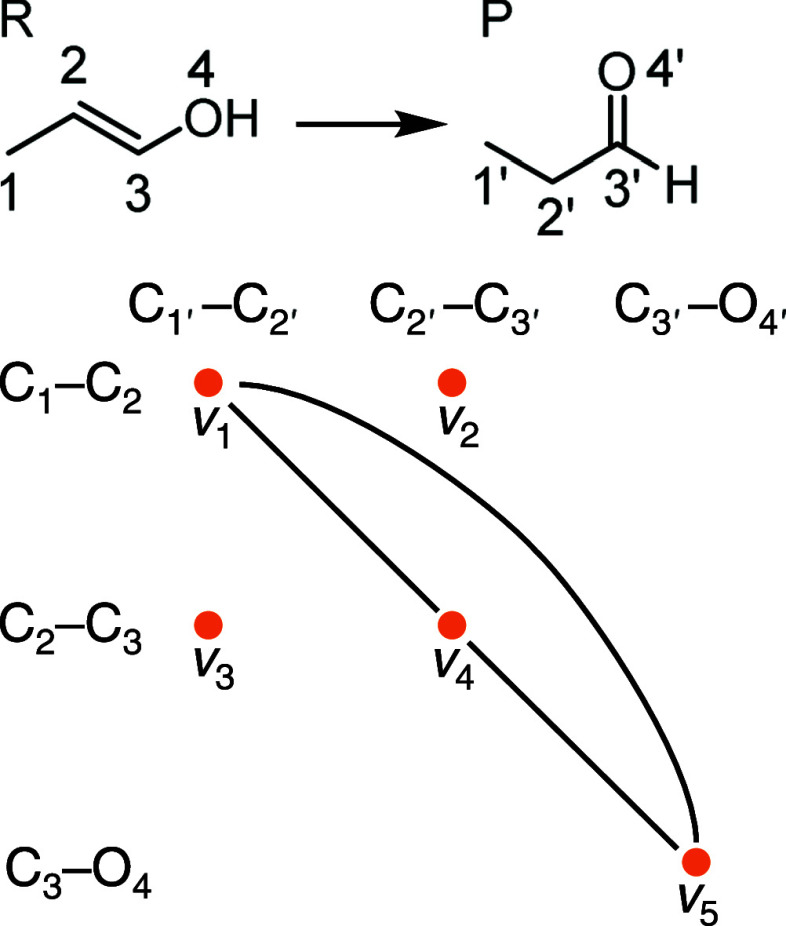
Modular product for the reaction from 2-methylvinyl alcohol (*R*) to 1-propanal (*P*). The vertices in the modular product represent pairs of chemical bonds of the same type, one from *R* and the other from *P*. Each pair of compatible vertices is connected by an edge in the modular product. (See the text for the detailed definition of the modular product.) This modular product contains one maximum clique, {*v*_1_, *v*_4_, *v*_5_}, which corresponds to the bond-to-bond correspondences C_1_–C_2_ ↔ C_1′_–C_2′_, C_2_–C_3_ ↔ C_2′_–C_3′_, and C_3_–O_4_ ↔ C_3′_–O_4′_. These three bonds are considered plausible unchanged bonds, resulting in the MCES with the C–C–C–O structure in *R* and *P*.

An established approach to compute MCESs is to search for the maximum cliques in the modular product^48,49^ of the reactant and product molecular graphs, *R* and *P*, as illustrated in Figure 2. Each vertex in the modular product represents a mapping from one bond in the reactants to one bond in the products; thus, a set of vertices defines a bond-to-bond correspondence. The MCESs can be regarded as the bond-to-bond correspondences between unchanged bonds in the reactants and products. As explained below, the MCESs are defined by the largest sets of vertices where every pair of vertices is connected by an edge, i.e., the maximum cliques of the modular product.

Each vertex in the modular product is a pair of chemical bonds of the same type, one from *R* and one from *P*, representing a one-to-one correspondence between them. Here, we define the type of a chemical bond by the element types of the atoms involved in the bond (e.g., chemical bonds C–C and C=C are considered the same type, but different from C–O, C–N, etc.). In our approach, we intentionally omit differences in bond multiplicity to avoid “overfitting” to the positions of double or triple bonds, which can shift during chemical transformations, such as in pericyclic reactions.^14,16^ Additionally, we ignore chemical bonds involving hydrogen atoms to reduce computational complexity. For instance, the modular product in Figure 2 contains four bond pairs of the C–C type (*v*_1_–*v*_4_) and one bond pair of the C–O type (*v*_5_) as its vertices.

Each edge of the modular product indicates the compatibility of the bond-to-bond correspondences (i.e., bond pairs) represented by its end point vertices. Two bond pairs (*b*_1_, *b*_1_^′^) and (*b*_2_, *b*_2_^′^) are called incompatible if and only if one of the following conditions is met: (1) chemical bonds *b*_1_ and *b*_2_ in the reactants are identical or chemical bonds *b*_1_^′^ and *b*_2_^′^ in the products are identical; (2) *b*_1_ and *b*_2_ share an atom of the reactants but *b*_1_^′^ and *b*_2_^′^ do not share any atom of the products, or vice versa. Condition (1) implies that the two bond-to-bond correspondences map the same bond in the reactants to different bonds in the products, or vice versa, meaning that the combination of these mappings does not define a one-to-one correspondence between bonds in the reactants and products. For example, in Figure 2, vertices *v*_1_ and *v*_2_ are disconnected because they map C_1_–C_2_ bond in the reactant to different bonds, C_1′_–C_2′_ and C_2′_–C_3′_, in the product. Condition (2) implies that the combination of the two mappings results in an atom-to-atom correspondence where the shared atom splits or merges during the reaction, which contradicts the principle of atom indivisibility. For instance, in Figure 2, vertices *v*_3_ and *v*_5_ are disconnected due to the condition (2)—the two mappings C_2_–C_3_ → C_1′_–C_2′_ and C_3_–O_4_ → C_3′_–O_4′_ would be possible only if the atom C_3_ in the reactant could split during the reaction. In contrast, every compatible bond pair is connected by an edge in the modular product.

A set of vertices in the modular product defines a feasible correspondence between bonds in the reactants and products if every pair of vertices is connected, i.e., all bond pairs are compatible with each other; this correspondence is one-to-one and does not contradict the principle of atom indivisibility. In graph theory, such a set of fully connected vertices is called a clique. As each vertex in a clique of the modular product represents the pair of identical bond between the reactants and products, the size of the clique equals the number of unchanged bonds. Since the MCESs maximize the number of unchanged bonds, the maximum cliques correspond to the MCESs. For instance, in Figure 2, the maximum clique of the modular product is {*v*_1_, *v*_4_, *v*_5_}, which corresponds to the MCES with the C–C–C–O structure in *R* and *P*. Note that in our definition of the modular product, where the bond multiplicity is ignored, the correspondence between the C=C and C–O bonds in *R* and the C–C and C=O bonds in *P* is considered valid. If the bond multiplicity is taken into account, the MCES would be much smaller, limited to the C–C structure.

The MCESs typically do not contain all atoms involved in the reaction, resulting in partial mappings between atoms in the reactants and products. These partial mappings can be completed by all possible permutations of the residual atoms for each element. In this way, we can compute all complete mappings through the maximum clique enumeration.

### 2.2 Clustering and Filtering Multiple AAM Solutions

All complete mappings obtained as AAM solutions contain some chemically equivalent mappings, which are generated by molecular symmetry in the reactants and products. Clustering these equivalent mappings by molecular symmetry yields chemically nonequivalent mappings that represent distinct reaction patterns. Our algorithm automatically clusters the complete mappings by molecular symmetry and returns a representative mapping from each cluster. (See Supporting Information for the mathematical details of the symmetry reduction method.)

**Figure 3:**
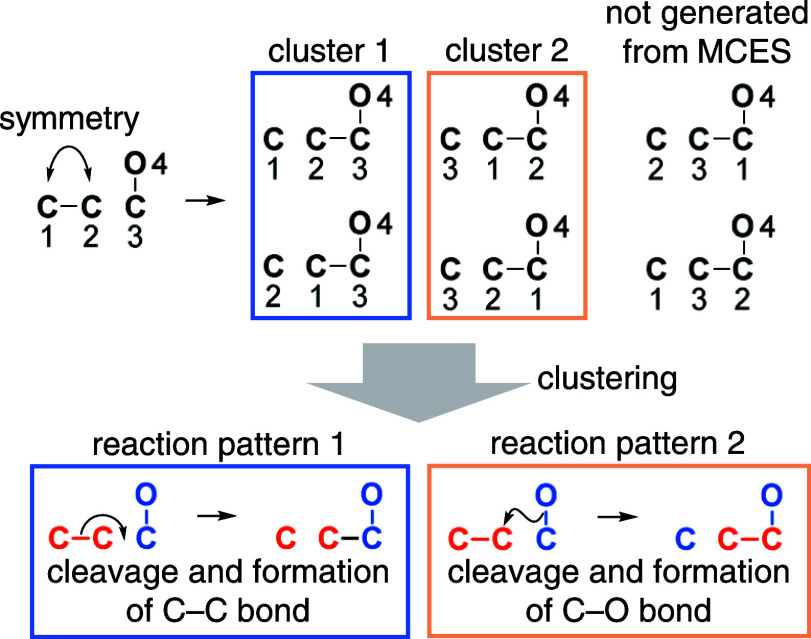
Clustering multiple AAM solutions by molecular symmetry. The virtual reaction presented in this figure has six possible mappings: four with one bond cleavage and one bond formation (shown in the left two columns, corresponding to the MCESs), while two with two bond cleavages and two bond formations (shown in the right columns, not corresponding to the MCESs). The four mappings can be clustered by the symmetry associated with the carbon atoms 1 and 2 in the reactant and classified into two distinct reaction patterns.

Figure 3 shows an example of the symmetry reduction for a virtual reaction. In this reaction, there are six possible mappings, which are generated by atom label permutation: four with one bond cleavage and one bond formation, while two with two bond cleavages and two bond formations. The first four mappings correspond to the MCESs and are considered plausible. These mappings include equivalent mappings generated by the symmetric carbons 1 and 2 in the reactant. They can be classified into two nonequivalent reaction patterns: one via C–C bond cleavage and formation, and the other via C–O bond cleavage and formation. Therefore, the number of nonequivalent mappings in this case is two.

Optionally, our algorithm can further reduce the number of AAM solutions using filters based on information about hydrogen atoms and bond multiplicity, which are neglected in the modular product defined in the previous subsection. In this optional postprocessing step, the algorithm selects complete mappings that (1) minimize the total number of created or broken bonds involving a hydrogen atom—we refer to this selection as filter1—and that (2) minimize the total number of bond multiplicity changes after applying filter1—we refer to this selection as filter2.

### 2.3 Enumerating All Maximum Cliques by Ising Computing

The maximum clique problem of graph *G* can be formulated as a quadratic unconstrained binary optimization (QUBO) problem
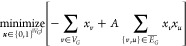
1where *V_G_* denotes the vertex set of *G*,  designates the complement of the edge set of *G*, and *A* is a positive constant parameter greater than one^1^. Each variable *x*_*v*_ indicates whether the vertex *v* ∈ *V*_*G*_ is included in a clique (*x*_*v*_ = 1) or not (*x*_*v*_ = 0). The first term of the objective function counts the number of vertices included in a clique (i.e., clique size, or the number of unchanged bonds), which should be maximized. In turn, the second term penalizes the situation that the selected vertices do not form a clique; it takes a positive value if and only if nonadjacent vertices *v* and *u* (i.e., ) are selected, which contradicts the definition of cliques. The constant parameter *A*(>1) controls the magnitude of the penalty term. In general, if the penalty strength is too large, the performance of SA will deteriorate; thus we set *A* to two, a moderate value greater than one, in this study. Because a QUBO problem is mathematically equivalent to an Ising model problem through the relation *x*_*i*_ = (1 – σ_*i*_)/2, we can solve the maximum clique problem by Ising computing.

In this research, we employed simulated annealing (SA)^34^ for solving the maximum clique problem formulated in eq 1. SA is a metaheuristic algorithm designed to solve NP-hard optimization problems, inspired by the annealing process in metallurgy. Several Ising computing devices are based on variants of SA.^51−55^

As an SA-based solver can be viewed as a sampler obeying a probability distribution close to the Gibbs distribution, an appropriate sampling strategy is demanded to enumerate all maximum cliques—corresponding the ground states of an Ising model—with high probability. In this study, we employed an enumeration algorithm utilizing Ising computing proposed by Mizuno et al.^29^ (see also the Supporting Information for the details). This enumeration algorithm aims to enumerate all ground states of an Ising model by repeating sampling solutions. Notably, this algorithm includes a parameter ϵ, which controls the failure rate of exhaustive enumeration with a theoretical guarantee under certain conditions (see the Supporting Information). These conditions have been empirically confirmed to hold for the benchmark problems used in this study. We set ϵ to 0.01, ensuring the success probability is over 99%.

## 3 Results and Discussion

This section presents the benchmark results of our proposed algorithm. To evaluate the performance of our proposed method, we used a benchmark data set of 241 reaction SMILES in a published paper after some corrections.^16^ This data set includes various reactions, such as fundamental reactions (S_N_2, E2, S_N_Ar, esterification, amidation, borylation, radical reaction, etc.), named reactions (Robinson cyclization, Diels–Alder reaction, Friedel–Crafts reaction, Larock indole synthesis, etc.), and protection and deprotection reaction (silylation of alcohol, acetalization of ketone, etc.). These reactions mainly consist of C, N, O, and no transition metals. In particular, Cl and Br are frequently contained as heavy atoms; F, Si, P, and S are sometimes included. Li, B, and Mg are also contained as other light elements. See the Supporting Information for the details of the benchmark setting.

First we will explore the computational advantages of the SA-based enumeration algorithm for solving maximum clique enumeration problem, which is an NP-hard combinatorial optimization problem, in comparison to an existing maximum clique enumeration algorithm in Section 3.1. Second we will examine the chemical benefit of our AAM algorithm, in comparison to existing AAM algorithms based on chemical heuristics in Section 3.2.

### 3.1 Computation Time for Maximum Clique Enumeration

First, we evaluated the computation time for our proposed algorithm to enumerate all maximum cliques in the modular product of a chemical reaction with a success probability over 0.99. We conducted a comparative analysis with a well-established exact algorithm for the maximum clique problem introduced by Carraghan and Pardalos,^56^ integrated with an efficient clique enumeration strategy developed by Bron and Kerbosch^57^ and later improved by Tomita, Tanaka, and Takahashi.^58^ We refer to this conventional algorithm as CP-exact algorithm hereinafter. The performance was evaluated by measuring the average computation time over 10 iterations for enumerating the maximum cliques in each corresponding chemical reaction. To mitigate the computational burden, we imposed a 2 h upper bound time limit for each case and evaluated both the CP-exact and SA-based enumeration algorithms on the benchmark data set. The tolerant failure rate of the Ising-computing-based-enumeration algorithm was set to 0.01 to ensure a success probability greater than 0.99.

Under the time limit, our SA-based enumeration algorithm successfully resolved 234 cases out of 241, whereas the CP-exact algorithm successfully resolved only 197 cases. We compared the maximum cliques generated by the SA-based method with those produced by the CP-exact algorithm for the 197 cases and found no discernible differences (i.e., all 10 instance runs of the SA-based method succeeded in finding the correct answer for each of the 197 cases).

**Figure 4:**
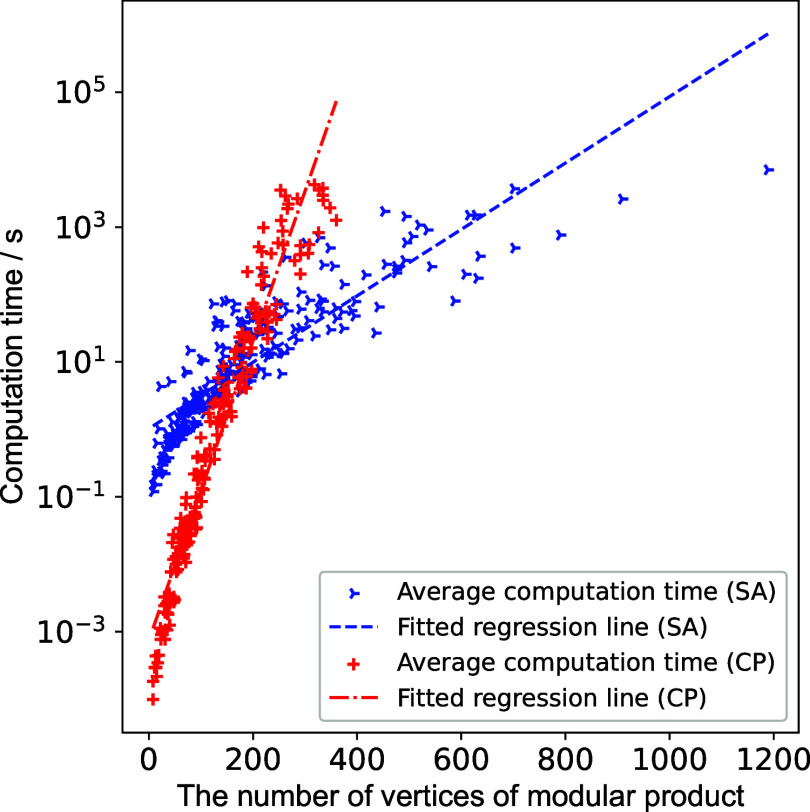
Computation time for SA-based maximum-clique-enumeration algorithm compared with the CP-exact algorithm.^56−58^

The computation times of both algorithms exhibited exponential increases with respect to the number of vertices in the modular product, denoted by *N* (see Figure 4). In comparison, our SA-based enumeration algorithm demonstrated a flatter slope than the traditional CP-exact algorithm and exhibited superior performance in cases with large *N*. Quantitatively, the computation time of the SA-based enumeration algorithm is approximately proportional to exp(0.011*N*), while that of the CP algorithm is proportional to exp(0.051*N*). This means that the former is expected to solve a problem with *N* = 1000 in 8.6 × 10^4^ s, while the latter takes 1.2 × 10^19^ s. Additionally, *N* correlates with the number of atoms involved in the chemical reaction, denoted by *n*. The relationship can be approximately expressed as *N* ≈ 0.21·*n*^2.26^ (see the Supporting Information). These approximation equations allows one to estimate the computation time required for the AAM of a chemical reaction and assess the applicability of our algorithm based on the number of atoms.

The computation time of the SA-based algorithm varied, even for problems with similar *N*. One factor contributing to this variation is the difference in the number of maximum cliques to be enumerated, denoted by *m*. This is because the number of required sampling times in the SA-based enumeration algorithm is expected to be *O*(*m* ln *m*).^29^ In general, reactions with higher molecular symmetry are expected to involve a larger number of maximum cliques, resulting in longer computation times for the SA-based enumeration.

In summary, the SA-based enumeration algorithm succeeded in finding the correct set of the maximum cliques in the AAM with high probability and with less computational complexity than the conventional algorithm, making the proposed AAM procedure with minimal chemical knowledge more practical.

### 3.2 Chemical Correctness of the AAM Solutions

**Figure 5:**
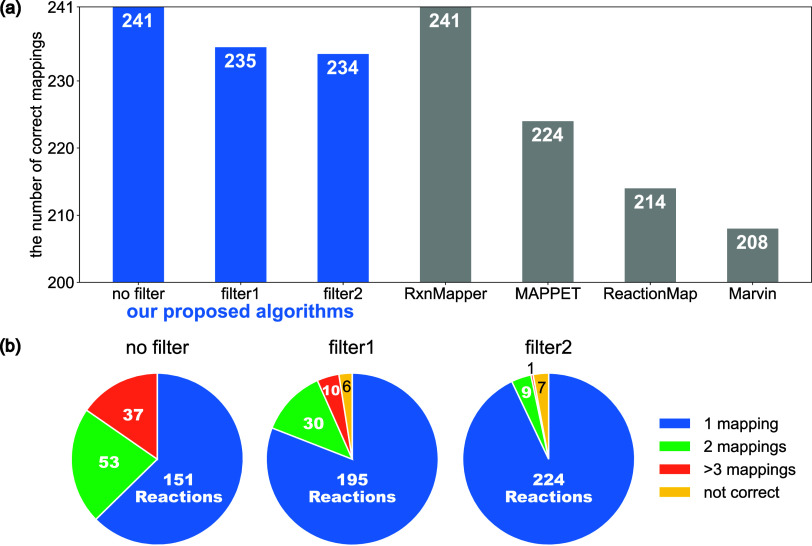
Chemical correctness of the AAM solutions for the 241 reactions data set. (a) The number of correct mappings computed by each algorithm. Here, “no filter” indicates the results for our proposed algorithm without any postfiltering, “filter1” minimizes the number of bond cleavages and formations involving a hydrogen atom, and a “filter2” minimizes the number of bond multiplicity changes after applying filter1. (b) The number of nonequivalent mappings for each option of our algorithm.

Next, we examined the chemical correctness of the AAM solutions generated by our algorithm by comparing them with expert-provided answers from the benchmark data set. In this examination, the time limit imposed in the previous subsection was not applied. Our algorithm succeeded in computing candidate atom mappings for all 241 reactions. We also compared the AAM results with four existing tools: RxnMapper,^23^ MAPPET,^16^ ReactionMap,^14^ and Marvin.^15^ The results of the chemical correctness comparison are presented in Figure 5.

Our proposed algorithm succeeded in finding the correct mappings for all 241 reactions without optional postfiltering (indicated as “no filter”). This performance is the same as the state-of-the-art machine learning model RxnMapper and better than the other three algorithms. In 151 out of 241 cases (63%), our enumeration algorithm identified correct and unique mappings without postfiltering. We also applied two types of optional filters: a filter minimizing the number of bond cleavages and formations involving a hydrogen atom (i.e., the number of hydrogen atoms moving during the reaction, filter1), and a filter minimizing the number of bond multiplicity changes after applying filter1 (filter2). Filter1 considerably increases the number of cases with unique correct nonequivalent mappings from 151 (63%) to 195 (81%) by filtering multiple nonequivalent mappings. This filtering process incorrectly excluded the correct mappings in only six cases. This result suggests that the number of hydrogen atoms moving during the reaction is an effective criterion for identifying chemically correct mappings in most reactions. Filter2 further increased the number of cases with unique correct nonequivalent mappings to 224 (93%), while increasing the number of failure cases by only one. Additionally, after applying the filters, our framework rarely returned three or more nonequivalent mappings in this data set.

**Figure 6:**
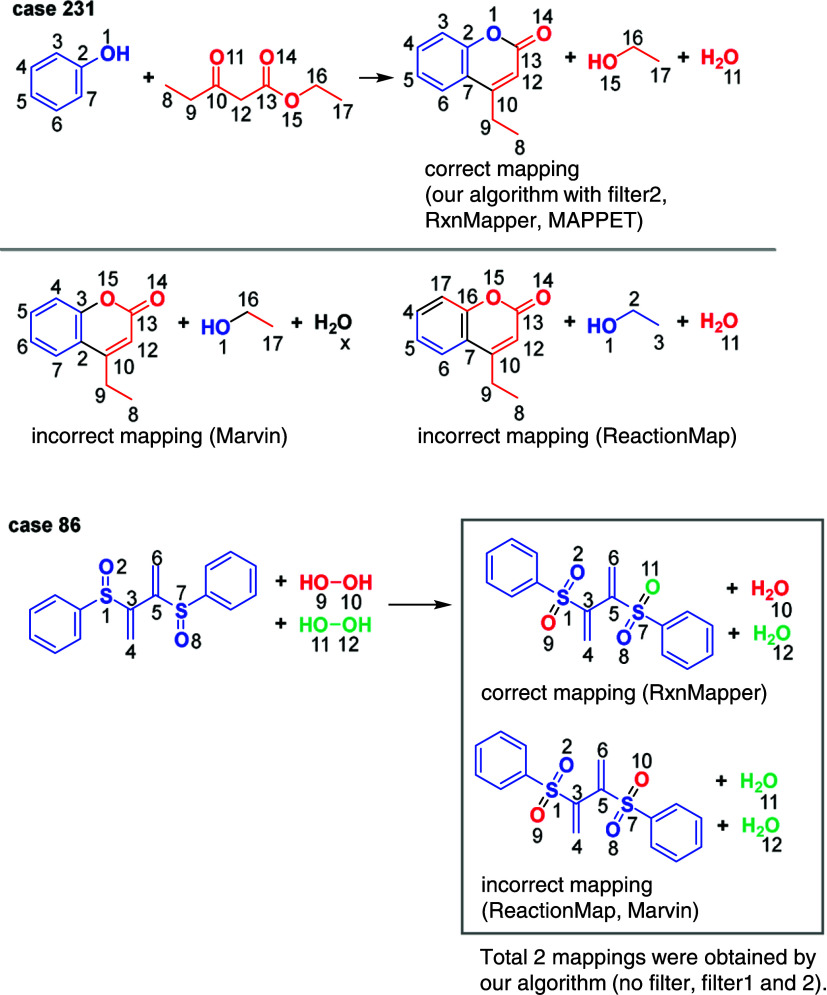
Case studies of AAM solutions in the data set. Trivial mapping numbers were omitted for clarity. Black bonds in the products represent those formed during the chemical transformation, and an *x* mark indicates the algorithm’s failure to determine a mapping for the corresponding atom.

The mappings generated by three existing tools—MAPPET, ReactionMap, and Marvin—are less accurate than those generated by our algorithm. For example, in Figure 6, for reaction case 231, our algorithm with filter1 generated a unique correct mapping, while ReactionMap and Marvin produced incorrect mappings. Especially, an unusual phenol ring decomposition was observed in the mapping generated by ReactionMap. These incorrect mappings involved three bonds cleavages, whereas the correct mapping involved only two bond cleavages. This case suggests that accurately calculating MCES (i.e., exactly minimizing the number of bonds broken and formed) is crucial for finding the correct mapping. As another example, in case 86, both ReactionMap and Marvin produced the same incorrect mapping. The assignment of oxygen atom’s origin in this mapping was incorrect. Our algorithm, both with and without filtering, generated two plausible mappings, including both the incorrect and correct mappings. This case shows the difficulty of determining the correct mapping by calculating only one MCES and emphasizes the importance of our enumerative approach.

**Figure 7:**
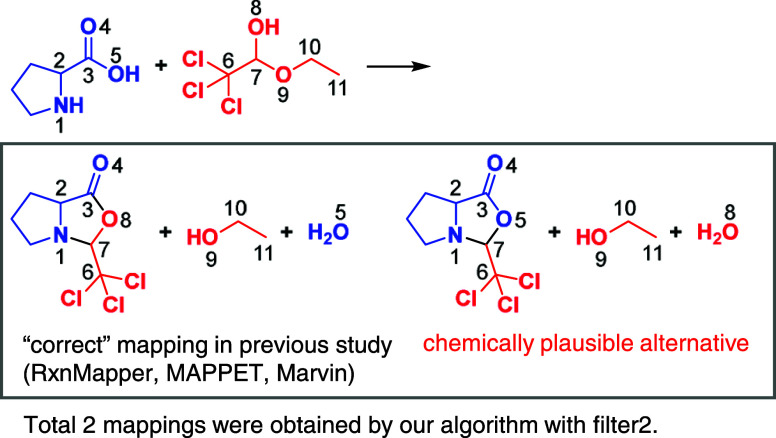
Possible candidate mappings for the reaction in case 22 from the benchmark data set.

The advantage of our enumeration algorithm is also highlighted in situations where it is difficult to determine which mapping is chemically correct even using expert chemical knowledge. This advantage is demonstrated in reaction 22 from the data set, as shown in Figure 7. Reaction 22 is the synthesis of oxazolidinone using the amino acid proline and ethyl chloral hydrate as substrates. Our proposed algorithm found two possible candidate mappings for this reaction with applying the aforementioned filter2. The difference between these mappings lies in the origin of the inner-ring oxygen of oxazolidinone: in the left case, it originates from the hemiacetal; in the right case, from the carboxylic acid. The original MAPPET paper^16^ of the benchmark data set considered the left, esterification-like mechanism to be correct, and other two algorithms—RxnMapper and Marvin—also provided the left mapping as solutions. However, several studies have reported that this type of reaction can proceed through a nucleophilic attack by the oxygen of the carboxylate in proline on the C=N bond of an iminium produced from the aldehyde (originating from the hemiacetal) and proline.^59,60^ Therefore, the right mechanism is also a chemically plausible alternative in this context, and further investigation may be required to determine which candidate mapping is actually correct. In this way, our algorithm generates possible candidate mappings, allowing chemists to determine the correct mapping by consulting the literature or conducting experiments, if necessary.

The state-of-the-art machine learning model RxnMapper provided the correct answers in all cases, showing the same performance as our algorithm. Moreover, the execution times of RxnMapper were very short, ranging from 0.025 to 0.088 s. In contrast, the execution times of our algorithm were much longer, primarily due to the computational demand of the NP-hard maximum clique enumeration, as shown in Figure 4. However, as discussed above, RxnMapper may miss alternative mappings that originate from uncommon reaction mechanisms, and it can overfit to more common reaction mechanisms such as esterification. Given these features, our AAM framework is expected to serve as an alternative tool for careful assessment of mappings, especially for reactions with unknown or uncommon mechanisms—which is our original motivation of this study.

## 4 Conclusions

We have developed an algorithm for exhaustively enumerating all plausible AAM solutions based on the MCESs in chemical reactions. Our framework incorporates an enumeration algorithm to identify all possible maximum cliques by SA-based Ising computing. This enumeration algorithm not only accurately identifies the maximum cliques corresponding to the MCESs but also significantly outperforms established exact algorithms for maximum clique enumeration in terms of computation time. Furthermore, in terms of chemical correctness, our proposed AAM algorithm surpasses most of existing AAM tools and exhibits the same performance as the state-of-the-art machine learning model RxnMapper. Additionally, it identifies multiple nonequivalent mappings in some cases, and these enumerated mappings include chemically correct reaction patterns. This advancement provides a valuable tool for chemoinformatics, enabling more precise and efficient investigations of reaction mechanisms in organic chemistry.

Our proposed algorithm demonstrates promising advantages, particularly in its ability to enumerate all possible mappings for a wide range of chemical reactions, as it does not rely on known reaction patterns. However, it is currently limited to chemical reaction formulas where the numbers of atoms of each element on the reactant and product sides are equal (commonly referred to as balanced reaction in chemoinformatics community). Furthermore, the enumeration algorithm still requires long computation times when the number of maximum cliques to be enumerated is large due to molecular symmetry. The future challenges are first, improving the algorithm to analyze unbalanced reactions; second, incorporating symmetry reduction techniques before computing the MCESs to reduce computational complexity and make the algorithm more practical for analyzing complex reactions; and finally, utilizing Ising-computing hardware to enhance the scalability of our enumeration algorithm. These improvements are expected to further expand the applicability of our algorithm in chemoinformatics and organic chemistry research.

## Data Availability

All data of corrected SMILES data set, computation times, and comparison of correctness with other algorithms are available as Supporting Information. Our atom-to-atom mapping source code is available at https://github.com/aki-27/AAM-Ising.

## Supporting Information Available

Description of the symmetry reduction method; description of the enumeration algorithm using Ising computing; description of the data preparation process including corrections of the original data set; description of the relation between the number of vertices in a modular product and the number of atoms involved in a chemical reaction, including a figure (PDF)
Corrected SMILES data set; computation time data; chemical correctness data (ZIP)

## Footnotes

1 The present QUBO formulation of the maximum clique problem differs from the previous studies,^50^ but rather is equivalent to the QUBO formulation of the maximum independent set problem proposed in ref (50). The formulation given in eq 1 is simpler than that used in the previous studies in terms of the number of variables and the number of terms of the objective function. According to ref (50), the penalty parameter *A*, which controls the magnitude of the penalty term, must satisfy *A* > 1.
