# Supplementary material for: Enumeration Approach to Atom-to-Atom Mapping Accelerated by Ising Computing
Source: J Chem Inf Model. 2025 Feb 2;65(4):1901–10. doi: 10.1021/acs.jcim.4c01871 (PMC11863377; doi:10.1021/acs.jcim.4c01871)
Supplement: Supplementary file 1 — ci4c01871_si_001.pdf [file ci4c01871_si_001.pdf]

# Supporting Information for "Enumeration Approach to Atom-to-Atom Mapping Accelerated by Ising Computing"

Mohammad Ali,[†,‡] Yuta Mizuno,[*,†,¶,§] Seiji Akiyama,[*,§,‖] Yuuya Nagata,[§,‖] and Tamiki Komatsuzaki[†,¶,§,⊥]

†*Graduate School of Chemical Sciences and Engineering, Hokkaido University, Kita 13, Nishi 8, Kita-ku, Sapporo, Hokkaido 060-8628, Japan*

‡*Statistics Discipline, Khulna University, Sher-E-Bangla Rd, Khulna 9208, Bangladesh*

¶*Research Institute for Electronic Science, Hokkaido University, Kita 20, Nishi 10, Kita-ku, Sapporo, Hokkaido 001-0020, Japan*

§*Institute for Chemical Reaction Design and Discovery, Hokkaido University, Kita 21, Nishi 10, Kita-ku, Sapporo, Hokkaido 001-0021, Japan*

‖*ERATO Maeda Artificial Intelligence for Chemical Reaction Design and Discovery Project, Hokkaido University, Kita 21, Nishi 10, Kita-ku, Sapporo, Hokkaido 001-0021, Japan*

⊥*SANKEN, Osaka University, 8-1 Mihogaoka, Ibaraki, Osaka 567-0047, Japan*

E-mail: mizuno@es.hokudai.ac.jp; s.aki@icredd.hokudai.ac.jp

## Symmetry reduction method

This section provides the mathematical foundation of the symmetry reduction method, which clusters complete mappings constructed from maximum common edge subgraphs into equivalent classes representing distinct reaction patterns.

First, let us define a symmetric operation with respect to a molecular graph $M$. Let $V_M$ and $E_M$ denote the sets of vertices (atoms) and edges (bonds) of $M$, respectively. In this study, we define a symmetric operation with respect to $M$ as an atom label permutation $\sigma\colon V_M \to V_M$ such that (1) it rearranges the label of an atom in $V_M$ to that of another atom of the same element in $V_M$ and (2) it does not change the graph topology (i.e., it is a graph automorphism). The second condition on the graph automorphism can be written as

$$E_M = \{\{\sigma(u), \sigma(v)\} | \{u, v\} \in E_M\}\,. \tag{S1}$$

This condition implies that the mapping $\sigma$ preserves the set of edges (bonds). In other words, $\sigma$ represents a transformation of the molecular graph without bond cleavage and formation.

For simplicity of notation, let $\mathcal{T}_\sigma$ denote the edge set transformation induced by a bijective vertex mapping $\sigma$, which is defined as

$$\mathcal{T}_\sigma\colon E \mapsto \{\{\sigma(u), \sigma(v)\} | \{u, v\} \in E\}\,. \tag{S2}$$

Here, $\sigma\colon V \to V'$ is a bijection between two vertex sets $V$ and $V'$, $E$ is a set of vertex pairs in $V$, and $\mathcal{T}_\sigma(E)$ represents a set of vertex pairs in $V'$. (We here consider both the cases of $V = V'$ and $V \neq V'$ for the sake of later discussion.) Using this notation, we can write the graph automorphism condition simply as $\mathcal{T}_\sigma(E_M) = E_M$ with $V = V' = V_M$.

The edge set transformation $\mathcal{T}_\sigma$ has the following properties:

$$\mathcal{T}_{\sigma_1 \circ \sigma_2} = \mathcal{T}_{\sigma_1} \circ \mathcal{T}_{\sigma_2}, \tag{S3}$$

$$\mathcal{T}_{\text{id}} = \mathcal{I}, \tag{S4}$$

$$\mathcal{T}_{\sigma^{-1}} = \mathcal{T}_\sigma^{-1}, \tag{S5}$$

where id and $\mathcal{I}$ are the identity operators of the vertex mapping and the edge set transformation, respectively. Additionally, in the first equation, $\sigma_1 \circ \sigma_2$ is assumed to be well-defined. These properties implies that the set of all symmetric operations with respect to $M$, denoted by $\Sigma_M$, is a group in terms of the composition operator $\circ$, i.e., the following conditions hold:

$$(\sigma_1 \circ \sigma_2) \circ \sigma_3 = \sigma_1 \circ (\sigma_2 \circ \sigma_3), \tag{S6}$$

$$\text{id} \in \Sigma_M, \tag{S7}$$

$$\sigma \in \Sigma_M \Rightarrow \sigma^{-1} \in \Sigma_M. \tag{S8}$$

Next, we define the equivalence relation between two complete mappings $m_1$ and $m_2$ with respect to the reactant graph $R$ as

$$m_1 \sim_R m_2 \overset{\text{def}}{\iff} \exists \sigma \in \Sigma_R \text{ s.t. } m_2 = m_1 \circ \sigma, \tag{S9}$$

where $\sim_R$ designates the equivalence relation, and $\Sigma_R$ is the set of all symmetric operations with respect to the reactant graph $R$. Since the symmetric operation $\sigma$ is a transformation of the molecular graph without bond cleavage and formation, the condition $m_2 = m_1 \circ \sigma$ implies that the two mappings $m_1$ and $m_2$ involve the (essentially) same set of bond cleavage and formation. Thus equivalent mappings $m_1$ and $m_2$ represent the same reaction pattern.

This relation is actually an equivalence relation in mathematical sense:

$$m_1 \sim_R m_1, \tag{S10}$$

$$m_1 \sim_R m_2 \implies m_2 \sim_R m_1, \tag{S11}$$

$$m_1 \sim_R m_2 \wedge m_2 \sim_R m_3 \implies m_1 \sim_R m_3. \tag{S12}$$

The first equation holds because the identity operator id $\in \Sigma_R$ satisfies the condition $m_1 = m_1 \circ \mathrm{id}$. The second equation is valid because $m_2 = m_1 \circ \sigma$ implies $m_1 = m_2 \circ \sigma^{-1}$ and $\sigma \in \Sigma_R$ implies $\sigma^{-1} \in \Sigma_R$. The third equation can be proven by the fact that $\exists \sigma_{12} \in \Sigma_R$ s.t. $m_2 = m_1 \circ \sigma_{12}$ and $\exists \sigma_{23} \in \Sigma_R$ s.t. $m_3 = m_2 \circ \sigma_{23}$ leads to $m_3 = m_1 \circ (\sigma_{12} \circ \sigma_{23})$ and $\sigma_{12} \circ \sigma_{23} \in \Sigma_R$.

The equivalence condition [Eq. (S9)] can be tested easily by using the following relation:

$$\exists \sigma \in \Sigma_R \text{ s.t. } m_2 = m_1 \circ \sigma \iff \mathcal{T}_{m_1}(E_R) = \mathcal{T}_{m_2}(E_R), \tag{S13}$$

where $E_R$ is the set of edges (bonds) in $R$. We note that the domain of the complete mappings $m_1$ and $m_2$ is the vertex set of $R$, so $\mathcal{T}_{m_1}(E_R)$ and $\mathcal{T}_{m_2}(E_R)$ are well-defined. The proof of this relation as follows: (1) Assume there exists a symmetric operation $\sigma \in \Sigma_R$ satisfying $m_2 = m_1 \circ \sigma$. Then

$$\begin{aligned} \mathcal{T}_{m_2}(E_R) &= \mathcal{T}_{m_1 \circ \sigma}(E_R) && \text{(S14)} \\ &= \mathcal{T}_{m_1} \circ \mathcal{T}_{\sigma}(E_R) && \text{(S15)} \\ &= \mathcal{T}_{m_1}(E_R). && \text{(S16)} \end{aligned}$$

(2) In turn, suppose $\mathcal{T}_{m_1}(E_R) = \mathcal{T}_{m_2}(E_R)$ holds. Applying $\mathcal{T}_{m_1^{-1}}$ from the left to the both sides, we get

$$\mathcal{T}_{m_1^{-1}} \circ \mathcal{T}_{m_1}(E_R) = \mathcal{T}_{m_1^{-1}} \circ \mathcal{T}_{m_2}(E_R). \tag{S17}$$

As $\mathcal{T}_{m_1^{-1}} \circ \mathcal{T}_{m_1} = \mathcal{I}$, we obtain

$$\mathcal{T}_{m_1^{-1} \circ m_2}(E_R) = E_R. \tag{S18}$$

Furthermore, $m_1^{-1} \circ m_2$ is an atom label permutation which maps the label of an atom in $R$ to that of another atom of the same element in $R$. These facts imply that $m_1^{-1} \circ m_2$ is a symmetric operation with respect to $R$. Denoting $m_1^{-1} \circ m_2$ by $\sigma$, we get

$$m_2 = m_1 \circ \sigma \quad \wedge \quad \sigma \in \Sigma_R. \tag{S19}$$

This concludes the proof.

Similarly, we define the equivalence relation between two complete mappings $m_1$ and $m_2$ with respect to the product graph $P$ as

$$m_1 \sim_P m_2 \overset{\text{def}}{\iff} \exists \sigma \in \Sigma_P \text{ s.t. } m_2 = \sigma \circ m_1, \tag{S20}$$

where $\sim_P$ designates the equivalence relation, and $\Sigma_P$ is the set of all symmetric operations with respect to the product graph $P$. This equivalence relation can also be tested by the equation $\mathcal{T}_{m_1^{-1}}(E_P) = \mathcal{T}_{m_2^{-1}}(E_P)$, where $E_P$ is the edge set of $P$. Note that the vertex set of $P$ is the domain of the *inverse* complete mappings $m_1^{-1}$ and $m_2^{-1}$.

Finally, we define the equivalence relation between two complete mappings $m_1$ and $m_2$ with respect to both reactant and product graphs as

$$m_1 \sim m_2 \overset{\text{def}}{\iff} \exists \sigma_R \in \Sigma_R, \exists \sigma_P \in \Sigma_P \text{ s.t. } m_2 = \sigma_P \circ m_1 \circ \sigma_R. \tag{S21}$$

By denoting $m_1 \circ \sigma_R$ by $m'$, we can rewrite the above condition as

$$m_1 \sim m_2 \iff \exists m' \in \mathcal{C} \text{ s.t. } m_1 \sim_R m' \wedge m' \sim_P m_2, \tag{S22}$$

where $\mathcal{C}$ denotes the set of all complete mappings. This condition is helpful to judge if

$m_1 \sim m_2$ in terms of the equivalence relations $\sim_R$ and $\sim_P$. In practice, we construct an equivalence relation graph where each vertex represents a complete mapping and each edge indicates either equivalence relation $\sim_R$ or $\sim_P$; then we judge that $m_1 \sim m_2$ if there is a path from $m_1$ to $m_2$ in the equivalence relation graph. Furthermore, a connected component of the equivalence relation graph corresponds to an equivalence class defined as

$$[m] \coloneqq \{m' \in \mathcal{C} \mid m' \sim m\}, \tag{S23}$$

where $m$ is a representative mapping of the equivalence class $[m]$. The set of all representative mappings is the output of our AAM framework.

# Enumeration algorithm using Ising computing

In our AAM algorithm, we incorporate an Ising computing-based enumeration algorithm[1] to enumerate all maximum cliques involved in the AAM process. The enumeration algorithm repeatedly samples solutions with low cost-function values (i.e., low energy values) for the maximum clique problem formulated as QUBO, using simulated annealing (SA). The SA process may sample nonoptimal solutions because SA is designed to sample solutions following a probability distribution close to the Gibbs distribution at a low temperature. Thus, the enumeration algorithm retains the set of solutions with the lowest energy obtained so far, denoted by $S$, and refreshes the set $S$ when a lower energy solution is sampled. Furthermore, the algorithm stops the sampling process if the set $S$ has not been updated within a certain time limit (see below), ensuring that no other optimal solutions remain unsampled. It is proven that if the sampling probability distribution $p$ satisfies the condition:

$$\forall x_1 \in X,\ \forall x_2 \in X,\ E(x_1) \leq E(x_2) \Rightarrow p(x_1) \geq p(x_2),$$

the algorithm successfully enumerate all optimal solutions with a probability greater than $1 - \epsilon$. Here, $X$ denotes the set of solutions, $E$ represents the energy function (cost function), and $\epsilon \in (0, \mathrm{e}^{-1.5})$ is a user-specified tolerance for the failure rate. For example, the Gibbs distribution satisfies the above condition, and we empirically confirmed that the above condition is met in the maximum clique problems for the benchmark reactions.

The algorithm can be described by the following steps:

1. Sample an initial solution for the given problem. Initialize a set $S$ so that it contains only the initial solution, and set a variable $\theta$ as the energy value of the initial solution. The variable $\theta$ retains the current lowest energy value to reject higher energy samples and determine if $S$ should be refreshed.

2. Initialize a variable $\tau$ to one and a variable $m$ to two. Here, $\tau$ represents the number of

samples taken. The variable $m$ is used in the stopping condition for sampling, which sets the time limit for collecting $m$ distinct solutions as $m \ln(m\kappa_2/\epsilon)$.

3. Repeat the sampling process while $\tau$ is less than $m \ln(m\kappa_2/\epsilon)$, where $\kappa_2$ is a constant determined by $\epsilon$ (e.g., if $\epsilon = 0.01$, $\kappa_2 \simeq 2.44$). During the sampling process:

   (a) if a solution with energy higher than $\theta$ is sampled, discard it;

   (b) if a solution with energy equal to $\theta$ is sampled, add it to $S$ and increment $\tau$ by one;

   (c) if a solution with energy lower than $\theta$ is sampled, refresh the set $S$ to contain only the sampled solution, update $\theta$ to the new energy value, and return to Step 2.

4. At $\tau = \lceil m \ln(m\kappa_2/\epsilon) \rceil$, decide whether to continue the sampling process:

   (a) if the number of solutions in $S$ is greater than or equal to $m$, increment $m$ by one and return to Step 3;

   (b) if the number of solutions in $S$ is less than $m$ (i.e., the time limit has been exceeded), stop the sampling and return the final solution set $S$.

# Preparation of the chemical reaction data set

The benchmark data set was taken from “241 typical reactions with full stoichiometry” in a training set of a published paper.[2] We used this data set after cleaning and correction. The cleaning contains unifying a format of reaction SMILES and completing information on implicit hydrogens. The numbers of implicit hydrogen were guessed by the number of existing chemical bonds and the valence of each element. We found some apparent incorrectness in this data set, so we have corrected them (an incorrectness of case 125 was already pointed out in a previous study[3]). All corrected points of the data set are shown below in Table SI and Figures S1 and S2. Also, the bond multiplicities and hydrogens in chemical reactions are omitted except for cases of explicit hydrogen with mapping when calculating their modular products. These corrected reaction SMILES were transformed into graph structures by the SMILES parser of CGRTools.[4]

The benchmark results of ReactionMap[5] and Marvin[6] were calculated by the websites below. The benchmark results of MAPPET were referred from a published paper[2] except for corrected reactions: 11, 125, 203, 244, 239, and 241 .

https://cdb.ics.uci.edu/cgibin/reactionmap/ReactionMapWeb.py

https://marvinjs-demo.chemaxon.com/latest/demo.htm

(Marvin JS 24.3.0 (re389bc4da912), Build Date 2024-07-17 20:38, Licensed To Chemaxon internal)

Table SI: Corrections in the benchmark data set

| case | incorrect point | correction |
| --- | --- | --- |
| 11 | missing explicit hydrogens | adding explicit hydrogens |
| 125 | using duplicated mapping numbers 3 | correcting duplicated numbers |
| 203 | using duplicated mapping numbers 1 | correcting duplicated numbers |
| 205 | existence of an incorrect product H–Mg–Br | fixing overall the product |
| 224 | using duplicated mapping numbers 7 and 8 | correcting duplicated numbers |
| 239 | missing one oxygen atom in the product, and misaligned mapping of the benzene ring | adding the one oxygen atom in the product, and fixing mapping |
| 241 | existing an extra water molecule in the reactant | deleting the water from the reactant, and remapping |

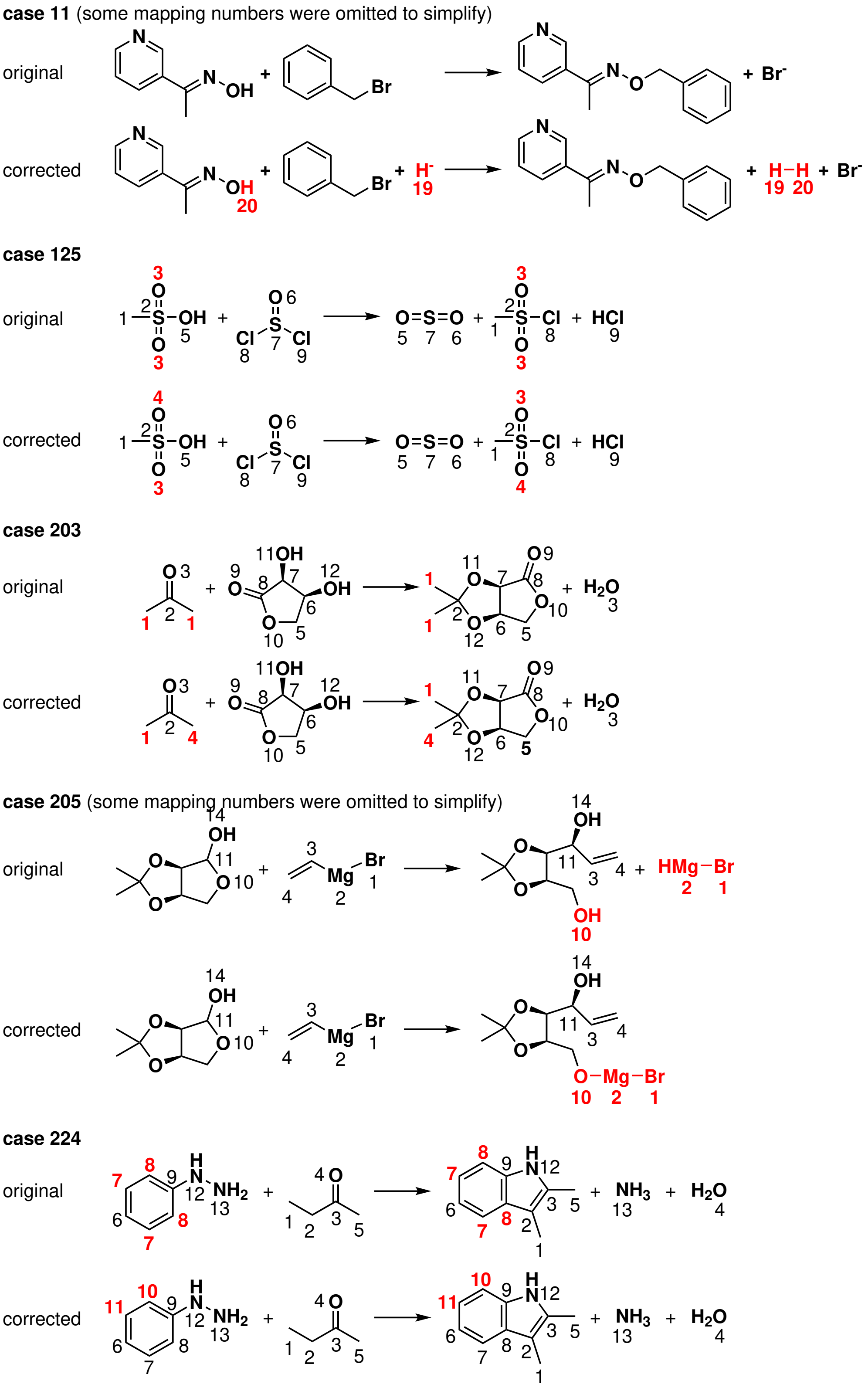


Figure S1: Corrections in the benchmark data set (case 11, 125, 203, 205, 224). Red parts indicate the incorrect and corrected points.

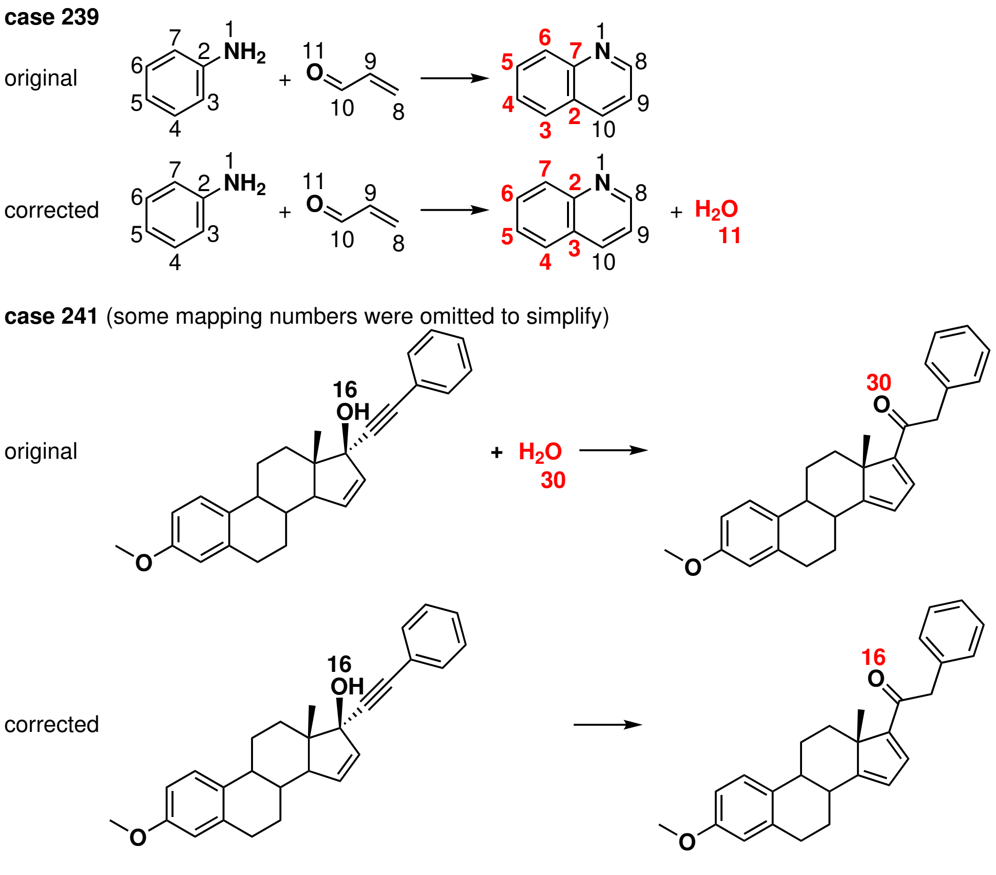


Figure S2: Corrections in the benchmark data set (case 239, 241). Red parts indicate the incorrect and corrected points.

# The number of vertices in a modular product vs. the number of atoms involved in a chemical reaction

We observed that the number of vertices in a modular product graph are associated with the number of atoms involved in the chemical reaction, as shown in Figure S3. The fitted regression line in the figure is given by $\ln N = -1.56 + 2.26 \ln n$, where $N$ is the number of vertices of the modular product and $n$ is the number of atoms. This relation enable us to estimate the modular product size from the number of atoms as $N \approx 0.21 \cdot n^{2.26}$. Note that the upper bound of $N$ is proportional to $n^2$ because the number of bonds is $\bar{d}n/2$ ($\bar{d}$: average vertex degree, typically less than four in organic chemistry) and its squared gives the upper bound of $N$. Furthermore, the value of the exponent may vary depending on the data set. Thus this approximation should be regarded as a rough estimation of $N$ for moderate $n$.

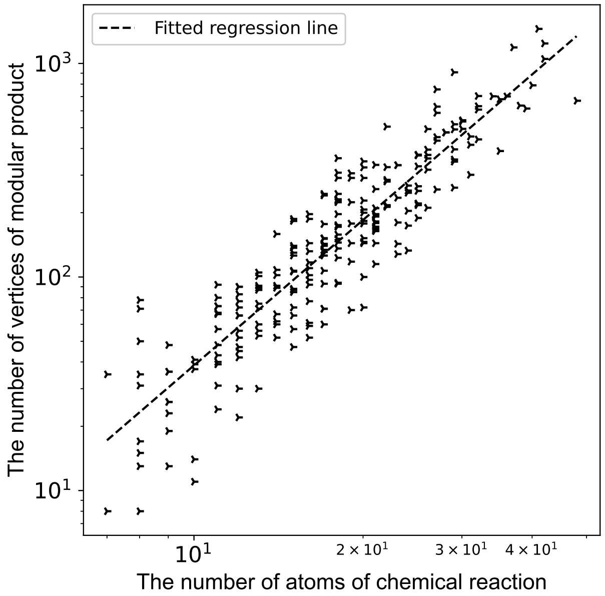


Figure S3: Relationship between the number of atoms involved in a chemical reaction (denoted by $n$) and corresponding number of vertices of the modular product graph (denoted by $N$). The relationship is approximated as $N \approx 0.21 \cdot n^{2.26}$.

# References


(1) Mizuno, Y.; Ali, M.; Komatsuzaki, T. Enumeration algorithms for combinatorial problems using Ising machines. 2024; `https://arxiv.org/abs/2412.00284`.

(2) Jaworski, W.; Szymkuć, S.; Mikulak-Klucznik, B.; Piecuch, K.; Klucznik, T.; Kaźmierowski, M.; Rydzewski, J.; Gambin, A.; Grzybowski, B. A. Automatic Mapping of Atoms Across Both Simple and Complex Chemical Reactions. *Nat. Commun.* **2019**, *10*, 1434.

(3) Lin, A.; Dyubankova, N.; Madzhidov, T. I.; Nugmanov, R. I.; Verhoeven, J.; Gimadiev, T. R.; Afonina, V. A.; Ibragimova, Z.; Rakhimbekova, A.; Sidorov, P.; others Atom-to-atom mapping: a benchmarking study of popular mapping algorithms and consensus strategies. *Mol. Inf.* **2022**, *41*, 2100138.

(4) Nugmanov, R. I.; Mukhametgaleev, R. N.; Akhmetshin, T.; Gimadiev, T. R.; Afonina, V. A.; Madzhidov, T. I.; Varnek, A. CGRtools: Python library for molecule, reaction, and condensed graph of reaction processing. *J. Chem. Inf. Model.* **2019**, *59*, 2516–2521.

(5) Fooshee, D.; Andronico, A.; Baldi, P. ReactionMap: An efficient atom-mapping algorithm for chemical reactions. *J. Chem. Inf. Model.* **2013**, *53*, 2812–2819.

(6) Cherinka, B.; Andrews, B. H.; Sánchez-Gallego, J.; Brownstein, J.; Argudo-Fernández, M.; Blanton, M.; Bundy, K.; Jones, A.; Masters, K.; Law, D. R.; Rowlands, K.; Weijmans, A.-M.; Westfall, K.; Yan, R. Marvin: A tool kit for streamlined access and visualization of the SDSS-IV MaNGA data set. *The Astronomical Journal* **2019**, *158*, 74.
